# Landscape of childhood and adolescent depression in Pakistan: experience from a tertiary care hospital in Karachi, Pakistan

**DOI:** 10.1192/bjo.2021.703

**Published:** 2021-06-18

**Authors:** Maria Khan, Mohummad Hassan Raza Raja, Fatima Gauhar, Tania Nadeem

**Affiliations:** The Aga Khan University Hospital

## Abstract

**Aims:**

Depression is highly prevalent in children and adolescents in Pakistan, yet, factors affecting depression have not been widely studied. This study aims to assess the demographic and clinical characteristics of depression in children and adolescents and identify associations between parental marital status and confounding factors for depression.

**Method:**

A descriptive retrospective study was undertaken at the Aga Khan University Hospital in Karachi, Pakistan. Patient records of children and adolescents (aged under 18 years), presenting to the psychiatry clinic with depression from 2015-2019 were reviewed. The diagnosis of clinical depression was made based on clinical assessment according to international guidelines. Patients whose medical records had missing information were excluded. Data were analysed using IBM SPSS Statistics for Windows, version 23.0 (IBM Corp., Armonk, N.Y., USA). Continuous data are presented as mean +/- standard deviation, whereas categorical data are presented as percentages (%). Pearson Chi-square test of association has been used to assess the association between parental factors and confounding factors. In instances where Pearson's Chi-square test could not be applied, Fisher's exact test is used instead. Associations at p <0.05 (95% confidence limit) are considered statistically significant.

**Result:**

A total of 133 participants were included, of which 78 (58.6%) were female, and 55 (41.4%) were male, with a mean age of 15.5 +/- 2.4 (Range: Ages 4–18). The population had a 50.4% prevalence of suicidal ideation, 21.1% of self-harm, 15% of substance abuse and 14.3% of suicide attempts. Academic stress (54.9%), inter-parental conflict (30.1%) and child abuse (29.3%) were the most common confounding factors reported. Other confounding factors include a family history of depression (20.3%), experience of bullying (16.5%) witnessing domestic violence (16.5%), substance abuse (15.0%) and experiencing sexual abuse (6.0%). There is a statistically significant association between children having parents with non-intact marriages and experiencing sexual abuse (p < 0.001, Odds Ratio (OR) = 21.48), having a family history of depression (p < 0.001, OR = 7.04), child abuse (OR = 3.78). Children of non-traditional (not living with both parents) families were more likely to witness domestic violence (p < 0.001, OR = 4.28), have a family history of depression (p < 0.001, OR = 3.44), abuse substances (OR = 3.20) and experience child abuse (OR = 2.48).

**Conclusion:**

This study identifies factors that may put children at an increased risk of developing depression and performing high-risk behaviours. The findings can help develop better screening programs and counselling for children and adolescents, allowing prevention and ensuring early diagnosis and care.

